# Machine learning enables automated screening for systematic reviews and meta-analysis in urology

**DOI:** 10.1007/s00345-024-05078-y

**Published:** 2024-07-10

**Authors:** H.S. Menold, V.L.S Wieland, C.M. Haney, D. Uysal, F. Wessels, G.C. Cacciamani, M.S. Michel, S. Seide, K.F. Kowalewski

**Affiliations:** 1 Department of Urology and Urological Surgery, University Medical Center Mannheim, University of Heidelberg, Theodor-Kutzer-Ufer 1-3, 68167 Mannheim, Germany; 2 Department of Urology, University of Leipzig, Leipzig, Germany; 3 Intelligent Systems and Robotics in Urology (ISRU), DKFZ Hector Cancer Institute at the University Medical Center Mannheim, Mannheim, Germany; 4 German Cancer Research Center (DKFZ), Heidelberg, Germany; 5 USC Institute of Urology, University of Southern California, ©, Los Angeles, CA USA; 6 Böhringer Ingelheim, Ingelheim am Rhein,, Germany

**Keywords:** Urology, Meta-analysis, Systematic reviews, Machine learning algorithms

## Abstract

**Purpose:**

To investigate and implement semiautomated screening for meta-analyses (MA) in urology under consideration of class imbalance.

**Methods:**

Machine learning algorithms were trained on data from three MA with detailed information of the screening process. Different methods to account for class imbalance (Sampling (up- and downsampling, weighting and cost-sensitive learning), thresholding) were implemented in different machine learning (ML) algorithms (Random Forest, Logistic Regression with Elastic Net Regularization, Support Vector Machines). Models were optimized for sensitivity. Besides metrics such as specificity, receiver operating curves, total missed studies, and work saved over sampling were calculated.

**Results:**

During training, models trained after downsampling achieved the best results consistently among all algorithms. Computing time ranged between 251 and 5834 s. However, when evaluated on the final test data set, the weighting approach performed best. In addition, thresholding helped to improve results as compared to the standard of 0.5. However, due to heterogeneity of results no clear recommendation can be made for a universal sample size. Misses of relevant studies were 0 for the optimized models except for one review.

**Conclusion:**

It will be necessary to design a holistic methodology that implements the presented methods in a practical manner, but also takes into account other algorithms and the most sophisticated methods for text preprocessing. In addition, the different methods of a cost-sensitive learning approach can be the subject of further investigations.

**Supplementary Information:**

The online version contains supplementary material available at 10.1007/s00345-024-05078-y.

## Introduction

Evidence-based medicine is the standard approach in modern patient care, incorporating clinical experience, patient will and current research findings. Systematic reviews (SRs) aim to collect and critically evaluate the complete evidence on a given topic in order to gain a better overview of the large amount of available data.

The different steps of SR and MA are highly standardized and defined according to the guidelines of the Cochrane Handbook for Systematic Reviews of Interventions [1] and by the Preferred Reporting Items for Systematic Reviews and Meta-Analysis (PRISMA) [2]. Overall, performing SR with or without MA is a time-consuming process that can involve more than 1,000 h [3]. In an analysis of 195 SR/MAs, Borah et al. showed that the average time to publication was more than one year (62 weeks), while some reviews took more than three years to be published [4].

Furthermore, during conduction of a SR/MA, only a fraction of all studies that are initially identified in the systematic literature search will be included in the final analysis. This is because the initial search is usually very broad to maximize sensitivity, as the costs of missing a relevant study might be worse than the higher workload during screening. This results in a classification problem, since the two classes (“included” and “excluded”) differ massively in number. This is also known as class imbalance. The use of machine learning (ML) algorithms has the potential to reduce the workload for research teams. However, ML algorithms tend to perform worse when class imbalance is present. To overcome this challenge, different measures to account for class imbalance such as sampling, cost-sensitive learning and weighting can be applied.

Sampling involves a modification of the training dataset. As such, upsampling can involve the random duplication or the synthetic generation of undersampled instances. Downsampling on the other side involves the random removal of the oversampled instances. As both methods result in a different dataset, the subsequently trained model will show a difference. Furthermore, cost-sensitive learning involves changing the cost of the model based on the errors made. As such, the cost of falsely excluding a study would be higher than the cost of falsely including a study. Weighting again involves a modification of the algorithm in which classes that are underrepresented are assigned higher weights in an attempt to address the present real-world considerations. In addition, classification thresholds can be shifted in either direction in order to obtain better results [5, 6]. Lange et al. provided a detailed comparison of methods in updating MA of diagnostic studies with respect to different ML or deep learning algorithms and also investigated the influence of initial text processing in detail [5]. However, a comprehensive analysis for an automated screening for SR/MA in urology, incorporating randomized and non-randomized trials with particular consideration of class imbalance does not exist.

Therefore, this study aimed to investigate the feasibility of an automated ML-based screening for meta-analysis comparing different algorithms and approaches to account for class-imbalance.

## Materials and methods

In the presented study, previously published MAs from our group were used to train the algorithms, as detailed information of the screening process (decision of two independent reviewers at each screening step) were available. The first review focused on the perioperative administration of blood transfusions during radical cystectomy [7]. The second review explored Radiomics for the classification of kidney tumors [8]. The third review, currently under publication, focuses on a critical analysis of the quality of evidence from randomized controlled trials (RCTs) in bladder cancer (PROSPERO: CRD42021247145) [9]. In the following, the three MAs are referred to as “Transfusion,” “Radiomics,” and “Urobase.”

### Machine learning algorithms

The systematic comparison of different ML algorithms is not the primary aim of the presented project work. However, to ensure that some adjustments for class imbalance are not dependent on the applied ML algorithm, three algorithms were examined. These algorithms were:

1. Random forest (RF)
2. Logistic regression with elastic net regularization (LogReg)
3. Support vector machines (SVM)

### Parameters and evaluation metrics

As input data for the models, different scenarios (see supplementary file 1, Table 1) were created, consisting of different combinations of available parameters (size of training dataset (200, 400, 600, 800 studies), consideration of the assessment of one human reviewer (include/exclude) and choice of text variables (abstract only, title + abstract, title + abstract + keywords). The data handling process is also described in the supplementary material.

The study aimed to solve a binary classification project, namely the decision (include / exclude) during the screening process of SR/Mas. Therefore, we assessed misses and work saved over sampling (WSS) as outcome parameters. WSS was introduced by Cohen et al. and is dedicated to be used for SRs [10]. For visualization purposes we created box-plots and violin plots as well as histograms. As performance parameters we used sensitivity, specificity and receiver operating characteristic (ROC)-curves with the area under the curve (AUC). Accuracy was not deemed adequate since due to the class imbalance problem, classifying all studies as “exclude” would result in high accuracy while still missing all relevant studies.

## Results

The characteristics of the three included reviews “Transfusion,” “Radiomics,” and “Urobase“ can be found in Table 1. While “Transfusion” and “Radiomics” are quite similar in the number of hits as well as the total number of hits found by the literature search, the systematic literature search for “Urobase” yielded significantly more hits. As another unique characteristic, only RCTs were considered for Urobase according to the study protocol. Additionally, the severe class imbalance becomes apparent (only 2–11% of all studies are included).

**Table 1:** Overview of the three included systematic reviews

	Transfusion [7]	Radiomics [8]	Urobase [9]
Number of studies found during literature search	992	882	5581
N° words: title (mean ± SD)	12.8 ± 4.7	13.7 ± 5.1	16.1 ± 6.7
N° words: abstract (mean ± SD)	226 ± 90	206 ± 81	237 ± 116
Token title	1,546	1,726	6,131
Token abstract	6,079	6,558	22,998
Token keywords	1,502	1,782	6,273
N° of studies „included“ after title/abstract screening (n (%))	38 (3.8)	102 (11.6)	295 (5.3)
N° of studies „included“ in final SR (n (%))	20 (2.0)	99 (11.2)	270 (4.8)

### Training process (on training data set)

As first step, ML algorithms were trained using the defined set of hyperparameters and input variables to define the optimal scenario (see supplementary file). The best model for each ML algorithm and sampling method was then used on the validation and test data set. This process was repeated for each of the three available SRs. As expected, for all reviews, integration of the choice of reviewer 1 (include/exclude), title + abstract + keywords, and larger size of the training data set yielded better results.

### Impact of different sampling methods on sensitivity

**Fig. 1:**
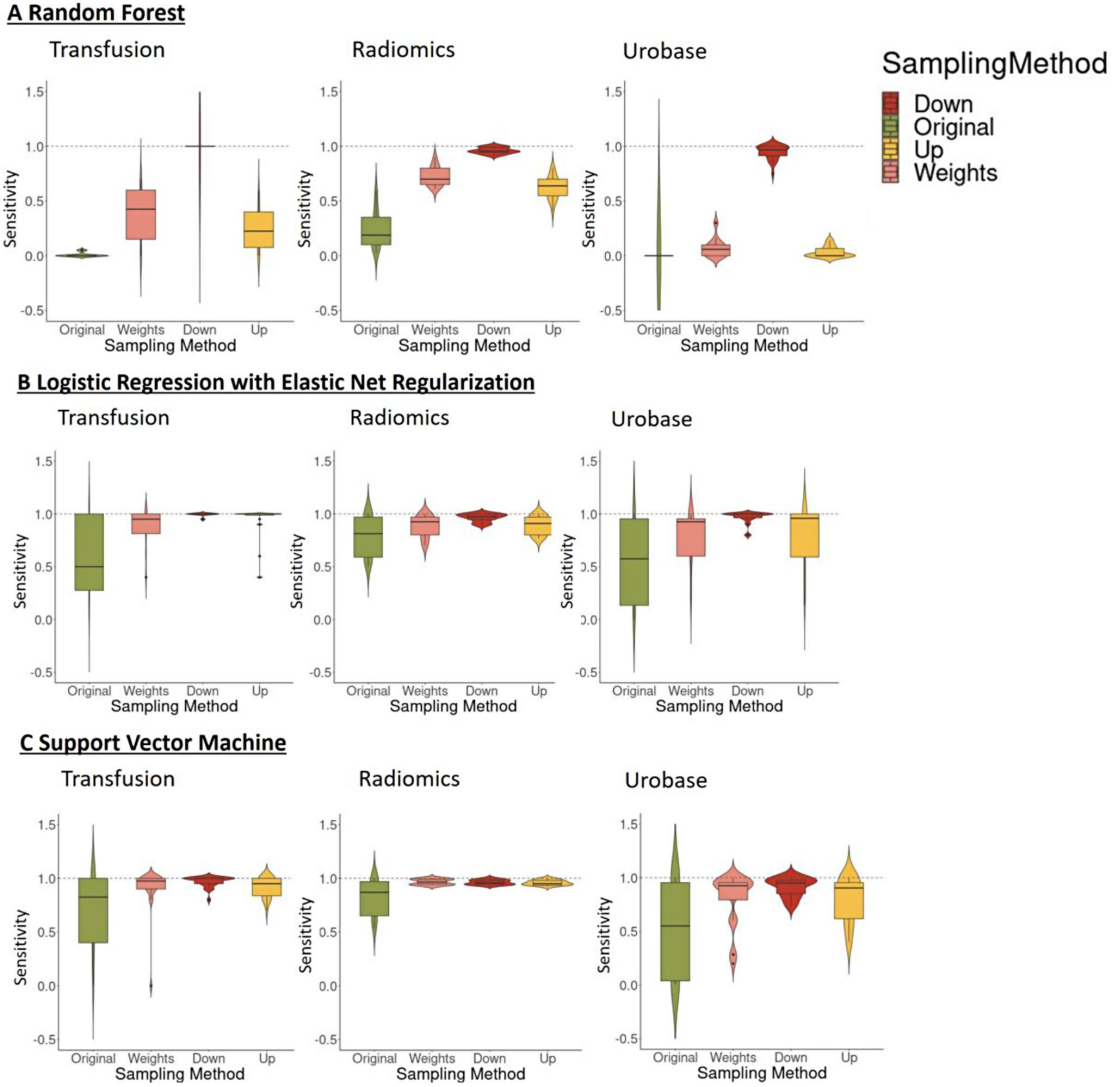
Performance comparison of different sampling methods based on sensitivity on training data set among the different MA/SRs

During model training, downsampling showed the best performance measured by the sensitivity. While there were no obvious differences between weighting and upsampling, the original data set had the worst sensitivity. In addition, downsampling showed the lowest dispersion which was consistent among all ML algorithms (see Fig. 1).

### Thresholding (on validation data set)

The selected models from the training data set, that performed best, were used on the validation data set. In a first step, ROCs with the respective AUCs were calculated (supplementary). The optimal threshold was then extracted as the threshold with a sensitivity of 1 with the best possible specificity. In addition further potential thresholds such as median and mean were extracted (see supplementary file).

### Final analysis (on test data set)

**Fig. 2:**
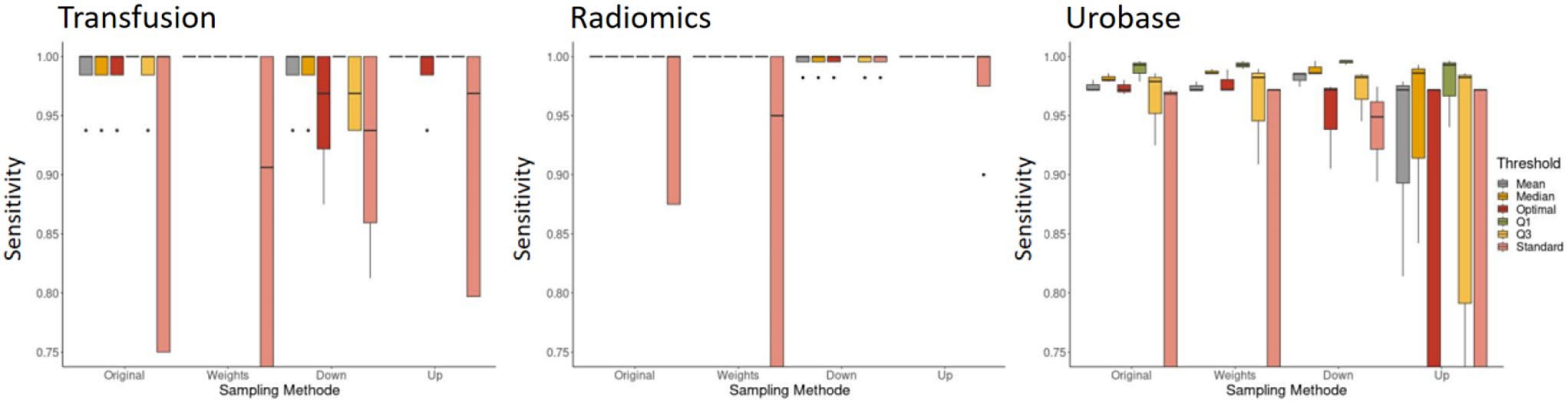
Final evaluation for sensitivity on test data set based on different thresholds and sampling methods

For the sensitivity as a decisive parameter for the given question, very good results were shown for the two SRs “Transfusion” and “Radiomics” as a sensitivity of 100% was achieved when the 1st quartile of all class probabilities from the test data set was selected as the threshold. For the “Urobase” SR, however, a worse performance was shown, with the 1st quartile of class probabilities as threshold again yielding the best sensitivity. Furthermore, the importance of thresholding is evident across all SRs examined, as the default threshold of 0.5 resulted in a decline in sensitivity. This was shown to be independent from the sampling approach. With regard to the sampling methods, the weighting approach proved superior to the other methods, with upsampling and downsampling yielding the worst performance in terms of sensitivity in the final test dataset (Fig. 2).

### Misses

**Fig. 3:**
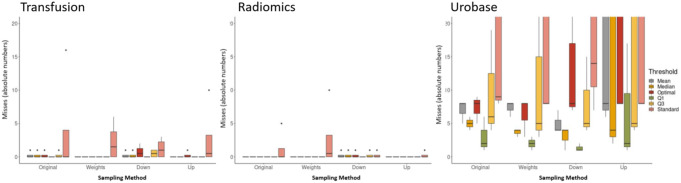
Overview of missed studies after title/abstract screening in relation to sampling method and threshold

As already evident from the sensitivity, some studies were missed by the models. Accordingly, the number of misses in the transfusion and radiomics SRs was low, whereas the number of misses in Urobase was significantly higher (Fig. 3). Similarly, it was confirmed that the weighting approach had the fewest misses. It is also clear that there is a need for thresholding, as many more studies would be missed with the standard threshold of 0.5. It must be noted that the misses shown in Fig. 5 refer to the title and abstract screening. Therefore, it was checked whether these misses were excluded in the full text screening anyway or whether they were finally also considered in the final SR. The number of misses of the respective final SR is shown in the supplementary material.

## Discussion

The aim of this work was to implement a method for a semi-automated title/abstract screening for SR/MAs in Urology with a focus on different measures to account for class imbalance. Overall the methods examined performed well in identifying relevant studies.

Regarding the different approaches, thresholding showed a positive impact on sensitivity, independent from sampling method or underlying review compared to the standard threshold of 0.5. Using diagnostic studies as an example, Ewald et al. were able to show that the choice of a data-driven threshold can lead to an increase in bias toward better test performance, especially when the prevalence is below 50% [11]. Accordingly, the definition of the optimal threshold depends on the evaluation metric under consideration [12].

Concerning the sampling methods, weighting was consistently shown to be superior to the other methods on the test dataset, across all reviews and the different thresholds. In contrast, during model training, there was a clear advantage of downsampling across all reviews as measured by the sensitivity. This underlines the importance of independent data sets for model training and testing. In previous works, van Hulse et al. showed that the performance of different sampling methods is sometimes dependent on the ML algorithm used [13]. Japkowicz et al. also compared different sampling methods in their study and concluded that both random upsampling and downsampling are effective methods for adjusting class imbalance [14]. In a recent study, Nishant et al. reported a hybrid method combining upsampling and downsampling. This method was shown to be superior to the other upsampling methods investigated in this study [15]. However, as our study suggests a weighting should be implemented based on the presented results.

The different methods to account for class imbalance are different mechanisms during model building, thus a combination of the methods is possible as presented in our study. Thresholding, as a post-hoc procedure, does not lead to any improvement of the algorithm or the model itself, but only the shifting of the threshold [16]. In contrast, sampling methods primarily belong to the preprocessing methods [17], since here the model training is based on a modified data set. Unlike thresholding, a cost-sensitive learning approach has the potential to result in an actual improvement at the level of the algorithm itself. Lopez et al. compared sampling methods, cost-sensitive learning, and a combination of both [18].

Recently, Large Language Models (LLMs) are being increasingly explored for various applications within the context of SR and MA. Gou et al. test ChatGPT-4 against human reviewers for an automated paper screening. They found that LLMs should be used as an adjunct rather then a replacement for human reviewers [19]. This is in line with the findings by Khraisha et al. who also emphasized that the results of LLMs should be interpreted with caution while they might be helpful certain review tasks [20].

As a further limitation it must be mentioned that for text preprocessing only a small selection of text procession measures were applied. However, the importance of text preprocessing has already been examined previously and was not the aim of the current study.

## Conclusions

We were able to show that weighting in combination with thresholding yielded the greatest improvements. In the future, it will be necessary to design a holistic methodology that implements the methods presented here in a practical manner, but also takes into account other algorithms and a wide variety of methods for text preprocessing.

## Electronic supplementary material

Below is the link to the electronic supplementary material.

Supplementary Material 1

Supplementary Material 2
