# Supplementary material for: Machine learning enables automated screening for systematic reviews and meta-analysis in urology
Source: World J Urol. 2024 Jul 10;42(1):396. doi: 10.1007/s00345-024-05078-y (PMC11236840; doi:10.1007/s00345-024-05078-y)
Supplement: Supplementary file 2 — Supplementary Material 2 [file 345_2024_5078_MOESM2_ESM.docx]

**SUPPLEMENTARY Material:**

## Machine learning enables automated screening for systematic reviews and meta-analysis in urology

### Depiction oft he Workflow


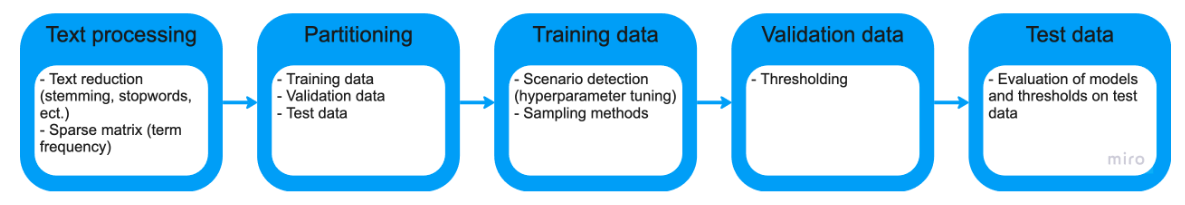


### 2. Project environment and programming

The project development and programming was done in R-Studio (R Version 3.6.2). Due to the high computational load, the final calculations were performed on an external server with 50 cores (R version 4.0.0, Platform x86_64-pc-linux-gnu, CentOS Linus 8). The results from the systematic literature searches from the above-mentioned SRs, including the reviewer decision during the screening steps were loaded into the R environment. Then, natural language processing was applied in order to prepare the datasets to be implemented in the ML algorithms. The *caret* package was used to implement different ML algorithms.

### 2. Working steps

The working steps were as follows (also see supplementary figure 1):

1. Text processing (text reduction techniques (replacing numeric enumerations in ordinal number words (e.g. “1.” to first), replacing dates with words, writing out abbreviations, transforming characters that were not in ASCII code, removing consecutive spaces, conversion to lowercase).
2. Tokenization (Transformation of text into R tidy format and tokenization (each word represents a token), removal stop words (e.g. “and”, “or”)
3. Creation of the sparse matrix (here the sparse matrix is a representation in which the matrix elements correspond to the number of each word in relation to the number of all words (term frequency) in the corresponding study)
4. Partitioning (Division into training data set (for model training and hyperparameter tuning), validation data set (for thresholding as post-hoc measure) and test data set for final analysis)
5. Modell training (implementation was done as described above with the caret package)
   - Re-Sampling method: Cross validation to 10-fold repetition
   - Calculation of *class probabilities* (for thresholding, standard value 0.5)
   - Method to account for class imbalance: Original data set, upsampling, downsampling, weighting
   - Implementation of different ML algorithims and their hyperparameters:
     - Random forest (package “range”): mtry, splitrule, min.node.size
     - LogReg (package “glmnet”): λ, α
     - Support Vector Machine (package „e1071“ with „svmLinear2“): cost
6. Thresholding (optimal threshold with sensitivity =1 and maximum specificity, as well as mean, median, Q1 and Q2 of all class probabilities)
7. Evaluation (see 2.3.)

### Different scenarios


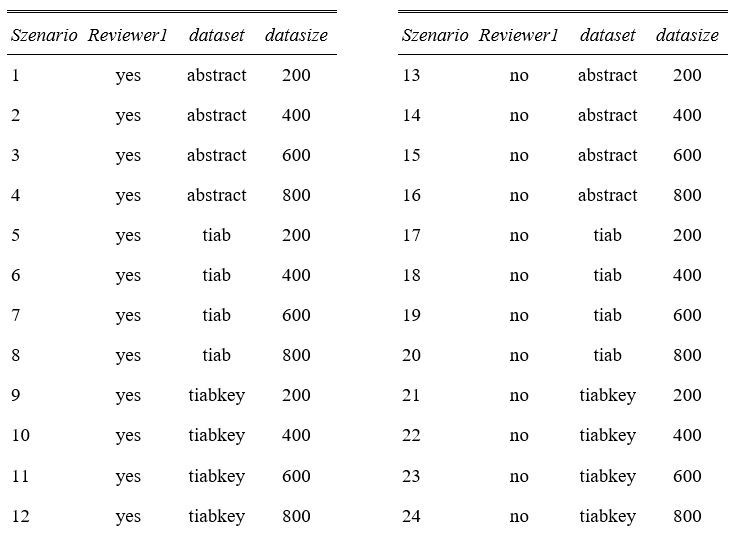


### 4. Scenario selection

**Table 1** Choice oft he optimal scenario (see above) during traing for each review [Original, Weighting, Downsampling, Upsampling]

| *Algorithmus* | ***Transfusion*** | ***Radiomics*** | ***Urobase*** |
| --- | --- | --- | --- |
| Random Forest | [23, 8 ,23 ,11] | [8, 8, 2, 4] | [4, 2, 2, 13] |
| LogReg | [11, 11, 11, 11] | [12, 12, 12, 12] | [9, 9, 2, 9] |
| Support Vector Machine | [9, 11, 10, 11] | [12, 12, 12, 12] | [9, 9, 9, 9] |

### Impact of choice of the other reviewer (inclusion / exclusion)

***Impact of additional variables on sensitivity***

When considering the choice of the other reviewer as potential variable, algorithms performed better. There was no clear advantage of using additional text inputs such as keywords and title in addition to the abstract text to improve sensitivity. This was also true for the size of the training data set (200 vs. 400 vs. 600 vs. 800 studies) with only marginal differences for sensitivity. The entire data is presented in the supplementary material.


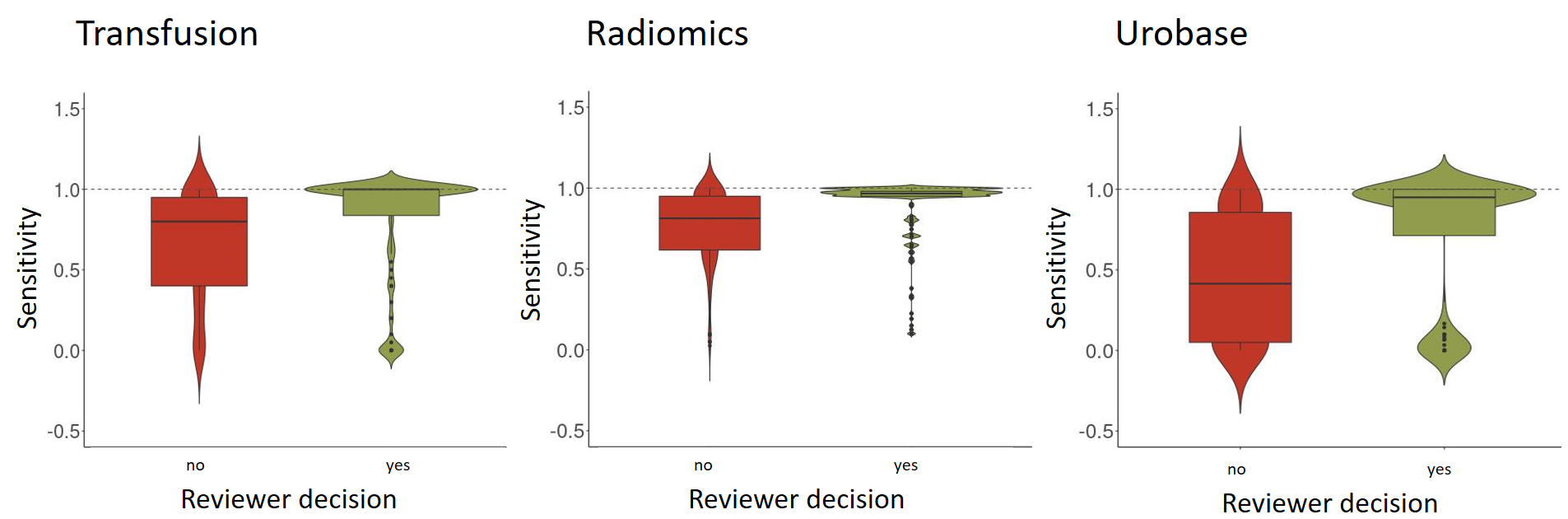


### 6. Impact text variables included as predictors (tiab = title + abstract; tiabkey= title + abstract + keywords)


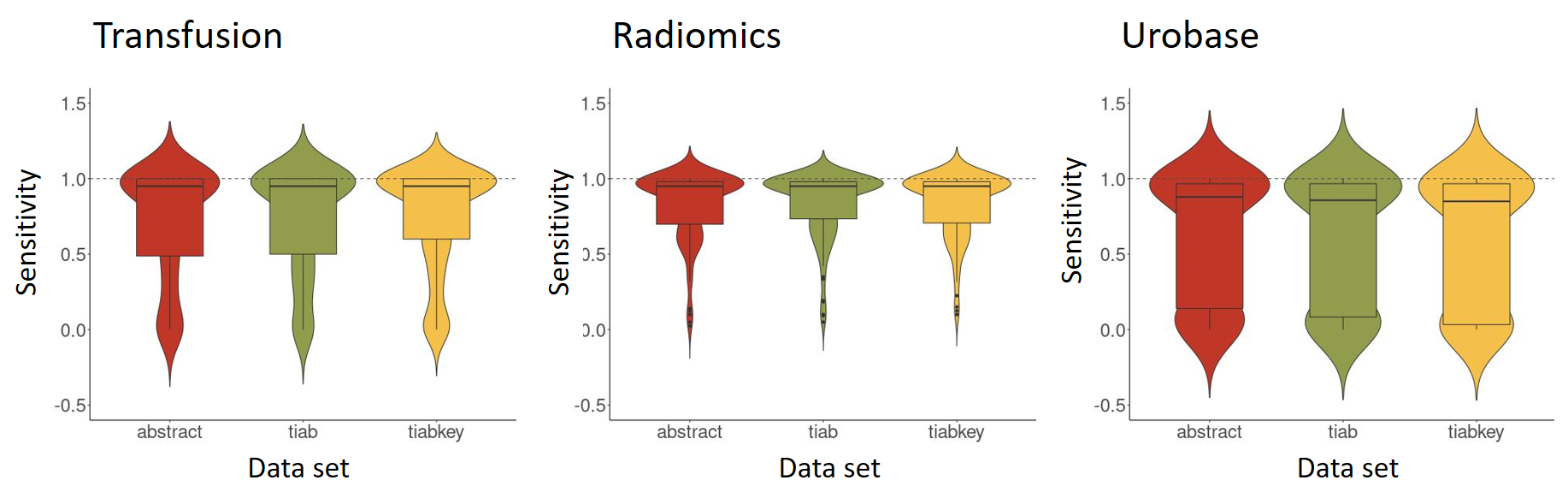


### 7. Impact number of studies used as training


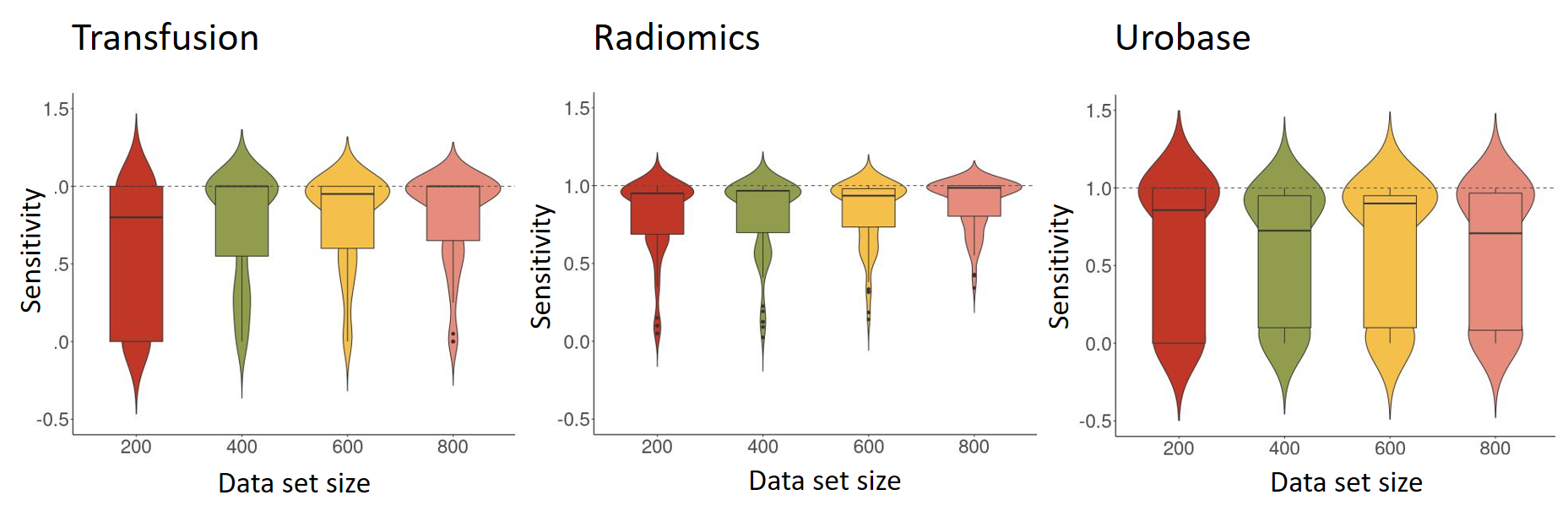


### 8. Computing time

The computing time to create the models based on algorithm and sampling method.

**Table 2** Computing time (seconds) for the creation of the models for each algorithm and sampling method

| *Algorithmus* | ***Transfusion*** | ***Radiomics*** | ***Urobase*** |
| --- | --- | --- | --- |
| Random Forest | [259, 267, 554, 677] | [322, 320, 617, 786] | [861, 864, 5677, 5834] |
| LogReg | [292, 273, 470, 776] | [337, 319, 557, 775] | [734, 681, 4627, 4915] |
| Support Vector Machine | [251, 299, 457, 573] | [294, 332, 527, 620] | [660, 759, 3779, 3847] |

### 9. AUC on validation set

Overviews AUCs on validation data set

| *Review* |  | *Algorithmus* | *Original* | *Weights* | *Down* | *Up* |
| --- | --- | --- | --- | --- | --- | --- |
| ***Transfusion*** |  | RF | 92,11 | 97,07 | 96,73 | 96,43 |
|  |  | LogReg | 99,93 | 98,07 | 93,60 | 99,55 |
|  |  | SVM | 100 | 99,85 | 100 | 99,85 |
| ***Radiomics*** |  | RF | 100 | 99,93 | 98,58 | 100 |
|  |  | LogReg | 99,29 | 99,57 | 99,40 | 99,53 |
|  |  | SVM | 99,78 | 99,69 | 99,69 | 99,80 |
| ***Urobase*** |  | RF | 91,01 | 89,68 | 97,92 | 94,71 |
|  |  | LogReg | 100 | 100 | 100 | 100 |
|  |  | SVM | 100 | 100 | 100 | 100 |

### 10. Thresholding on validation data set for the different MA/SR

Transfusion SR

| ***RF*** | *Original* | *Weights* | *Down* | *Up* |
| --- | --- | --- | --- | --- |
| Optimal | 0,02283333 | 0,15748208 | 0,48304667 | 0,16810064 |
| Q1 | 0,00566667 | 0,04750747 | 0,36855990 | 0,03705706 |
| Median | 0,01700000 | 0,08150487 | 0,41550449 | 0,07664828 |
| Mean | 0,03345392 | 0,10626176 | 0,42024894 | 0,10213422 |
| Q3 | 0,03440000 | 0,14134678 | 0,46447609 | 0,14664425 |
| ***LogReg*** |  |  |  |  |
| Optimal | 0,45610677 | 0,14578587 | 0,41470418 | 0,26665636 |
| Q1 | 0,00055804 | 0,04439012 | 0,29183292 | 0,06412245 |
| Median | 0,00055804 | 0,05921567 | 0,34994534 | 0,07602288 |
| Mean | 0,03822904 | 0,10137396 | 0,36801670 | 0,12052788 |
| Q3 | 0,00055804 | 0,08904118 | 0,43169558 | 0,10811793 |
| ***SVM*** |  |  |  |  |
| Optimal | 0,31288503 | 0,41159795 | 0,50806671 | 0,49674991 |
| Q1 | 0,00766952 | 0,00252717 | 0,02117615 | 0,00248803 |
| Median | 0,00783498 | 0,00258374 | 0,03091589 | 0,00252984 |
| Mean | 0,02689796 | 0,03598465 | 0,06233232 | 0,04229319 |
| Q3 | 0,00815232 | 0,00264260 | 0,03206151 | 0,00258084 |

Radiomics SR

| ***RF*** | *Original* | *Weights* | *Down* | *Up* |
| --- | --- | --- | --- | --- |
| Optimal | 0.27182201 | 0.40776517 | 0.49157291 | 0.36655500 |
| Q1 | 0.01706667 | 0.06235152 | 0.42270391 | 0.04966984 |
| Median | 0.03888889 | 0.10285065 | 0.44684802 | 0.08506909 |
| Mean | 0.10652558 | 0.18621046 | 0.46829491 | 0.15794926 |
| Q3 | 0.09640012 | 0.21933095 | 0.49064442 | 0.18073870 |
| ***LogReg*** |  |  |  |  |
| Optimal | 0.03983621 | 0.15806554 | 0.16324328 | 0.15111525 |
| Q1 | 0.03701450 | 0.08189859 | 0.10635907 | 0.08168041 |
| Median | 0.03838687 | 0.10173880 | 0.12450061 | 0.10205590 |
| Mean | 0.10871443 | 0.19286147 | 0.21058168 | 0.19201367 |
| Q3 | 0.04053738 | 0.14662779 | 0.16890984 | 0.14905389 |
| ***SVM*** |  |  |  |  |
| Optimal | 0.00215112 | 0.14534084 | 0.01607128 | 0.00215442 |
| Q1 | 0.00208976 | 0.07513306 | 0.01535473 | 0.00208067 |
| Median | 0.00211059 | 0.14319455 | 0.01561201 | 0.00210562 |
| Mean | 0.10789260 | 0.13680857 | 0.11987418 | 0.11083625 |
| Q3 | 0.00213639 | 0.14435704 | 0.01591198 | 0.00213545 |

UROBASE SR

| ***RF*** | *Original* | *Weights* | *Down* | *Up* |
| --- | --- | --- | --- | --- |
| Optimal | 0.05013458 | 0.46465747 | 0.52161314 | 0.49508161 |
| Q1 | 0.03715070 | 0.46085672 | 0.42762061 | 0.48643791 |
| Median | 0.04981986 | 0.46768940 | 0.45673458 | 0.48930702 |
| Mean | 0.05195174 | 0.46889824 | 0.46465895 | 0.48971814 |
| Q3 | 0.06268622 | 0.47495858 | 0.50339234 | 0.49307541 |
| ***LogReg*** |  |  |  |  |
| Optimal | 0.51626132 | 0.65162377 | 0.57956073 | 0.64480232 |
| Q1 | 0.00086259 | 0.12210928 | 0.13649398 | 0.11635914 |
| Median | 0.00086259 | 0.12791444 | 0.14220435 | 0.12025551 |
| Mean | 0.04538033 | 0.16481500 | 0.19483374 | 0.16185808 |
| Q3 | 0.00086259 | 0.13355467 | 0.17157980 | 0.13466126 |
| ***SVM*** |  |  |  |  |
| Optimal | 0.47423738 | 0.39055847 | 0.43976024 | 0.49482146 |
| Q1 | 0.00851742 | 0.00761206 | 0.35847810 | 0.00748555 |
| Median | 0.00867793 | 0.00771330 | 0.38873243 | 0.00760717 |
| Mean | 0.05115985 | 0.04294399 | 0.37576031 | 0.05193610 |
| Q3 | 0.00883326 | 0.00780796 | 0.39095296 | 0.00773639 |
| ***Standard*** | 0.50000000 | 0.50000000 | 0.50000000 | 0.50000000 |

### 11. Work saved over sampling

***Work Saved over Sampling***

For WSS, there was no clear influence of the sampling method. Overall, there was a wide spread of the WSS. As expected, a lower threshold (e.g. Q1) saves less work for the reviewers. Depending on the data distribution of the class probabilities (see above), for example, in the case of a strongly skewed distribution for which Q1 = Q3, a large time saving could also be achieved for Q1 (Figure 4).


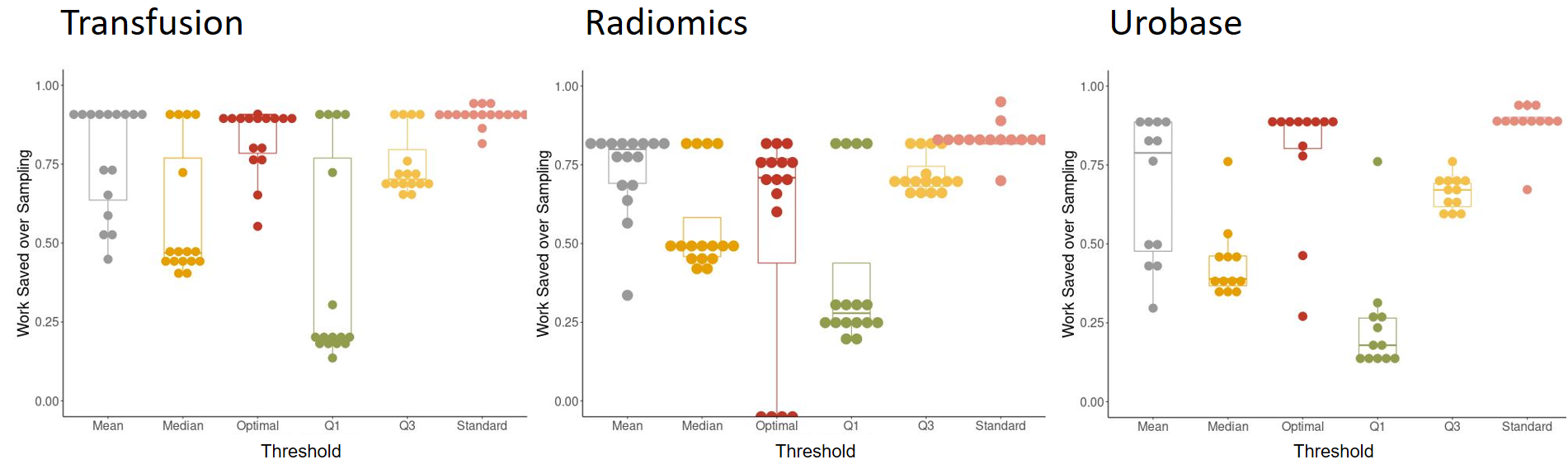


Overview for WSS in relation to different trheshold (the dots represent different sampling methods in combination with the applied ML algorithms).

### 12. Full text misses for all SR/MAs

*Transfusion:*

| ***RF*** | *Original* | *Weights* | *Down* | *Up* |
| --- | --- | --- | --- | --- |
| Optimal | 0 | 0 | 0 | 1 |
| Q1 | 0 | 0 | 0 | 0 |
| Median | 0 | 0 | 0 | 0 |
| Mean | 0 | 0 | 0 | 0 |
| Q3 | 0 | 0 | 0 | 0 |
| Standard | 9 | 3 | 0 | 4 |
| ***LogReg*** |  |  |  |  |
| Optimal | 0 | 0 | 0 | 0 |
| Q1 | 0 | 0 | 0 | 0 |
| Median | 0 | 0 | 0 | 0 |
| Mean | 0 | 0 | 0 | 0 |
| Q3 | 0 | 0 | 0 | 0 |
| Standard | 0 | 0 | 0 | 0 |
| ***SVM*** |  |  |  |  |
| Optimal | 0 | 0 | 0 | 0 |
| Q1 | 0 | 0 | 0 | 0 |
| Median | 0 | 0 | 0 | 0 |
| Mean | 0 | 0 | 0 | 0 |
| Q3 | 0 | 0 | 0 | 0 |
| Standard | 0 | 0 | 0 | 0 |

*Radiomics:*

| ***RF*** | *Original* | *Weights* | *Down* | *Up* |
| --- | --- | --- | --- | --- |
| Optimal | 0 | 0 | 0 | 0 |
| Q1 | 0 | 0 | 0 | 0 |
| Median | 0 | 0 | 0 | 0 |
| Mean | 0 | 0 | 0 | 0 |
| Q3 | 0 | 0 | 0 | 0 |
| Standard | 5 | 1 | 0 | 1 |
| ***LogReg*** |  |  |  |  |
| Optimal | 0 | 0 | 0 | 0 |
| Q1 | 0 | 0 | 0 | 0 |
| Median | 0 | 0 | 0 | 0 |
| Mean | 0 | 0 | 0 | 0 |
| Q3 | 0 | 0 | 0 | 0 |
| Standard | 0 | 0 | 0 | 0 |
| ***SVM*** |  |  |  |  |
| Optimal | 0 | 0 | 0 | 0 |
| Q1 | 0 | 0 | 0 | 0 |
| Median | 0 | 0 | 0 | 0 |
| Mean | 0 | 0 | 0 | 0 |
| Q3 | 0 | 0 | 0 | 0 |
| Standard | 0 | 0 | 0 | 0 |

*Urobase:*

| ***RF*** | *Original* | *Weights* | *Down* | *Up* |
| --- | --- | --- | --- | --- |
| Optimal | 3 | 2 | 16 | 144 |
| Q1 | 1 | 1 | 0 | 16 |
| Median | 3 | 3 | 1 | 43 |
| Mean | 3 | 6 | 1 | 50 |
| Q3 | 14 | 21 | 7 | 102 |
| Standard | 253 | 213 | 7 | 220 |
| ***LogReg*** |  |  |  |  |
| Optimal | 2 | 1 | 1 | 1 |
| Q1 | 0 | 0 | 0 | 0 |
| Median | 0 | 0 | 0 | 0 |
| Mean | 1 | 1 | 1 | 1 |
| Q3 | 0 | 0 | 1 | 0 |
| Standard | 2 | 1 | 1 | 1 |
| ***SVM*** |  |  |  |  |
| Optimal | 1 | 1 | 1 | 1 |
| Q1 | 0 | 0 | 0 | 0 |
| Median | 0 | 0 | 0 | 0 |
| Mean | 1 | 1 | 0 | 1 |
| Q3 | 0 | 0 | 0 | 0 |
| Standard | 1 | 1 | 21 | 1 |
