# Supplementary material for: Machine learning enables automated screening for systematic reviews and meta-analysis in urology
Source: World J Urol. 2024 Jul 10;42(1):396. doi: 10.1007/s00345-024-05078-y (PMC11236840; doi:10.1007/s00345-024-05078-y)
Supplement: Supplementary file 1 — Supplementary Material 1 [file 345_2024_5078_MOESM1_ESM.docx]

**Point-by-point Response**

## **Research Article:** Machine learning enables automated screening for systematic reviews and meta-analysis in urology

*We thank the editors and the reviewers for their valuable time and effort. We believe that due to the review of our work by peers we have been able to greatly improve the manuscript and believe that this has made it more valuable to the readership. All comments were addressed and changes are highlighted in the manuscript.*

*We will be at your disposal for any additional question*.

Reviewer 1:

Q1: Would NLP (natural language processing) and large language models (LLM) could be used to also help evaluating studies in SRs and MAs?

**A1: We thank the reviewer for this question.**

**Concerning your valuable comment with regard to LLMs, we added a paragraph to our discussion as we believe, as this is an interesting aspect for the readership. Thank you for this advice! p11, ll 30-34: “*Recently, Large Language Models (LLMs) are being increasingly explored for various applications within the context of SR and MA. Gou et al. test ChatGPT-4 against human reviewers for an automated paper screening. They found that LLMs should be used as an adjunct rather then a replacement for human reviewers [19]. This is in line with the findings by Khraisha et al. who also emphasized that the results of LLMs should be interpreted with caution while they might be helpful certain review tasks under specific conditions [20].*”.**

**Parts of NLP were already included and a necessary step in loading and editing the abstract for further handling. This is explained in detail in the supplementary material (Headline 2, subheading 1 &2). Here methods such as tokenization, stemming and other text reduction techniques were applied). We kept this in the supplementary material in order to not exceed the word count of our manuscript.**

Reviewer 2:

C1: Please, clearly define the purpose of the study in the introduction section.

**A1: Thank you. This was done accordingly in the end of the introduction with a clear goal of our study (page 5, lines 1-2) : “*Therefore, this study aimed to investigate the feasibility of an automated ML-based screening for meta-analysis comparing different algorithms and approaches to account for class-imbalance.”***

C2: Recently, generative AI based on large language models (LLMs) has been actively researched and should be discussed in the discussion section.

**A2: Thank you for this important remark! Please also refer to Question 1 of Reviewer 1. We added an paragraph to our discussion p11, ll 30-34: *“Recently, Large Language Models (LLMs) are being increasingly explored for various applications within the context of SR and MA. Gou et al. test ChatGPT-4 against human reviewers for an automated paper screening. They found that LLMs should be used as an adjunct rather then a replacement for human reviewers [19]. This is in line with the findings by Khraisha et al. who also emphasized that the results of LLMs should be interpreted with caution while they might be helpful certain review tasks under specific conditions [20].”***

**General note:** Some sentences of minor importance were deleted during the revision process. These are of course also marked in the manuscript.
